# The Anticancer Plant Triterpenoid, Avicin D, Regulates Glucocorticoid Receptor Signaling: Implications for Cellular Metabolism

**DOI:** 10.1371/journal.pone.0028037

**Published:** 2011-11-21

**Authors:** Valsala Haridas, Zhi-Xiang Xu, Doug Kitchen, Anna Jiang, Peter Michels, Jordan U. Gutterman

**Affiliations:** 1 Department of Systems Biology, University of Texas M. D. Anderson Cancer Center, Houston, Texas, United States of America; 2 Department of Computer-Aided Drug Discovery, Albany Molecular Research, Inc., Albany, New York, United States of America; 3 Department of Metabolism and Biotransformations, Albany Molecular Research, Inc., Albany, New York, United States of America; Wayne State University, United States of America

## Abstract

Avicins, a family of apoptotic triterpene electrophiles, are known to regulate cellular metabolism and energy homeostasis, by targeting the mitochondria. Having evolved from “ancient hopanoids,” avicins bear a structural resemblance with glucocorticoids (GCs), which are the endogenous regulators of metabolism and energy balance. These structural and functional similarities prompted us to compare the mode of action of avicin D with dexamethasone (Dex), a prototypical GC. Using cold competition assay, we show that Avicin D competes with Dex for binding to the GC receptor (GR), leading to its nuclear translocation. In contrast to Dex, avicin-induced nuclear translocation of GR does not result in transcriptional activation of GC-dependent genes. Instead we observe a decrease in the expression of GC-dependent metabolic proteins such as PEPCK and FASN. However, like Dex, avicin D treatment does induce a transrepressive effect on the pro-inflammatory transcription factor NF-κB. While avicin's ability to inhibit NF-κB and its downstream targets appear to be GR-dependent, its pro-apoptotic effects were independent of GR expression. Using various deletion mutants of GR, we demonstrate the requirement of both the DNA and ligand binding domains of GR in mediating avicin D's transrepressive effects. Modeling of avicin-GR interaction revealed that avicin molecule binds only to the antagonist confirmation of GR. These findings suggest that avicin D has properties of being a selective GR modulator that separates transactivation from transrepression. Since the gene-activating properties of GR are mainly linked to its metabolic effects, and the negative interference with the activity of transcription factors to its anti-inflammatory and immune suppressive effects, the identification of such a dissociated GR ligand could have great potential for therapeutic use.

## Introduction

**Figure 1:**
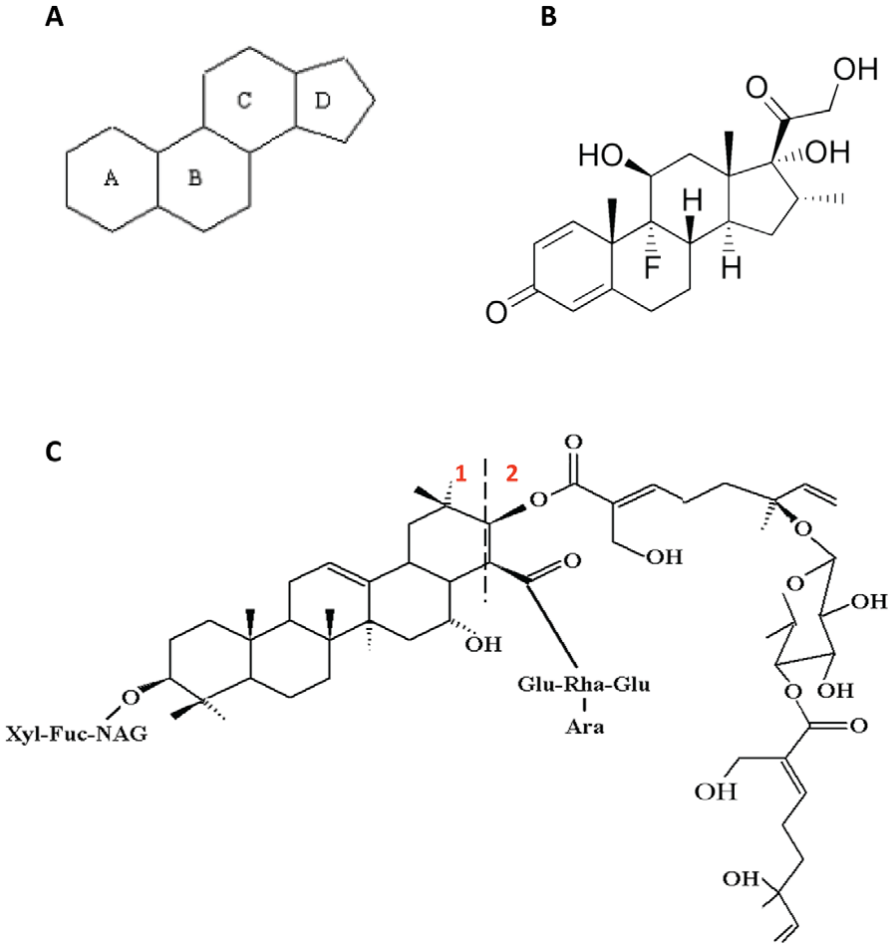
Chemical structures of steroids and avicin D. (A) The basic ring structure of a steroid molecule. (B) Chemical structure of dexamethasone, a prototypical steroid. (C) Chemical structure of avicin D. Part 1 of the molecule has the core 5-ring structure which resembles the core structure of a steroid molecule, and part 2 has a side chain containing two units of acyclic monoterpenes, connected by a quinovose sugar.

Avicins represent a family of triterpenes isolated from the seed pods of an Australian tree called *Acacia victoriae* [1]. Avicins were first identified by their ability to selectively induce apoptosis in various human tumor cells by direct perturbation of the mitochondria [2]. By targeting the mitochondria, avicins induce profound effects on cellular metabolism, which include lowering of oxygen consumption and ATP production [3], closing the voltage dependent anion channel (VDAC) [3], activation of AMPK [4], inhibition of mTOR [4] and induction of autophagy [4]. These effects induced by avicins result in a hypometabolic state. Other studies have also revealed avicins' ability to inhibit NF-κB [5] and activate NF-E2-related factor 2 (Nrf2) [6], accounting for their anti-inflammatory [5], [7] and stress responsive properties [6]. The stress regulatory properties of avicins could also be accounted for by their ability to lower cellular metabolism [2]–[4]. Avicins have evolved from ancient five ring triterpene structures called “hopanoids”, which are believed to be the precursors of sterols and formed the main membrane-lipid support in several prokaryotes, before oxygen was introduced into the atmosphere. Fig. 1 shows that the pentacyclic backbone in the avicin molecule resembles the four ringed core structure of the glucocorticoids (GCs). Based on (a) the structural similarity to (GCs), (b) the sensitivity of various GC responsive myeloma cells to avicin D [8], and (c) the fact that avicins regulate energy metabolism like GCs [4], we wanted to evaluate how avicins might interact with the GCs.

The results reported here demonstrate that avicins can bind to the glucocorticoid receptor (GR), and induce its nuclear translocation. However, avicin-induced nuclear translocation of GR does not lead to induction of GR-dependent transcription, but does cause inhibition of GR-driven NF-κB activity, suggesting that avicin D could have elements of a dissociated GR ligand. Modeling of avicin-GR interaction revealed that the avicin molecule binds to the antagonist confirmation of GR, supporting the hypothesis that avicin D could be a dissociated GR ligand. Avicin D, a pentacyclic terpene could therefore be classified as nonsteroidal selective GR modulator.

## Materials and Methods

### Cells

A549 (human lung carcinoma), HEK293T (human embryonic kidney) and HepG2 (human hepatocarcinoma) cells were all purchased from ATCC. A549 and HEK 293 T cells were cultured in DMEM supplemented with 10% FBS and 2 mM L-glutamine. Hep G2 cells were grown in alpha-MEM supplemented with 10% FBS and 2 mM L-glutamine.

### Cytokines and Reagents

Recombinant human TNF was purchased from BD Biosciences (San Jose, CA). Cold and radio labeled dexamethasone ([^3^H]-Dex) were purchased from Sigma (St. Louis, MO) and Amersham (Piscataway, NJ) respectively. Anti-phospho GR (Ser 211) and anti-total GR antibodies were a kind gift from Dr. M. Garabedian (New York School of Medicine, NY). Anti-PEPCK and anti-actin antibodies were purchased from Santa Cruz Biotechnology (Santa Cruz, CA). Anti-FASN antibodies were bought from Cell Signaling (Beverly, MA). Dual luciferase assay kit was bought from Promega (Madison, WI). Human interleukin-6 (IL-6) ELISA kit was purchased from R&D Systems (Minneapolis, MN).

### Plasmids

p(GRE)_2_-50huIL6P-luc+ and p(IL6κB)3-50huIL6P-luc+ plasmids were purchased from the BCCM/LMBP plasmid collection, Department of Molecular Biology, Ghent University, Belgium. Plasmids containing the wild type GR and GR deletion variants were a generous gift from Dr. Ronald. M. Evan (The Salk Institute for Biological Studies, La Jolla, CA).

### Transfection and Assay of Luciferase activity

HEK 293T cells were transiently transfected with luciferase reporter gene constructs, or plasmids carrying different forms of GR, using the Fugene transfection reagent from Roche (Indianapolis, IN), according to the manufacturer's protocol. TK renilla was always co-transfected with the luciferase reporters, for use as an internal control. 48 hrs post transfection, cells were treated with avicin D, Dex or RU486, as specified for each experiment. At the end of the treatment, cells were lysed in passive lysis buffer. Luciferase activity was measured in whole cell lysates, using the dual-luciferase assay kit (Promega) according to the manufacturer's protocol.

### Whole cell-Binding Assay

Cold competition binding assays were done as per Bamberger's protocol [9]. A549 cells were incubated with [^3^H]-Dex (25 nM) in the absence or presence of excess of unlabeled Dex or avicin D, for 1 hr at 37°C. At the end of the incubation, cells were washed three times with cold PBS, scrapped and resuspended in 100 µl of PBS. The cell suspension was added to 4 ml scintillation fluid, and radioactivity counted in a β-scintillation counter.

### Immunofluorescence Staining

549 cells were treated with avicin D (1 µM) for 0–60 min. Cells treated with Dex (1 µM, 60 min) were used as a positive control. At the end of the treatment, cells were washed twice with PBS, and fixed with 4% paraformaldehyde. The cells were permeabilized using 1% Triton X-100 and 0.5% NP40. After blocking the cells with 1% BSA, they were incubated with anti-GR antibody, followed by secondary (Rhodamine) antibody. Cells were then stained with 4′, 6-diamidino-2-phenylindole (DAPI) to permit visualization of nuclear DNA. The immunofluorescence was visualized by using a Leica DM LB fluorescence microscope. Images were captured with an automatic imaging system.

### Western blot analysis

Whole cell lysates were prepared from untreated and treated cells. Cellular proteins (50 µg) were resolved on an SDS polyacrylamide gel. Protein bands were detected by chemiluminescence (ECL, Amersham Pharmacia).

### Study of avicin D-GR docking

Avicin-D warhead was docked into two separate crystal structures for glucocorticoid: 1M2Z and 1NHZ (http://www.rcsb.org). The 1M2Z is the agonist version of the ligand binding domain (co-crystallized with dexamethasone) while 1NHZ is the antagonist form of the receptor (co-crystallized with RU-486). All docking calculations were performed with the Glide 5.7.109 docking algorithm (Glide, version 5.7, Schrödinger, LLC, New York, NY, 2011.). In all cases the protein preparation step was used prior to building the grids using the standard user interface. A docking box of 31 Å was defined around the co crystallized ligands. Default parameters were used for all docking calculations using the extra precision mode (Glide-XP).

## Results

### Avicin D interacts with GR, and induces its nuclear translocation

**Figure 2:**
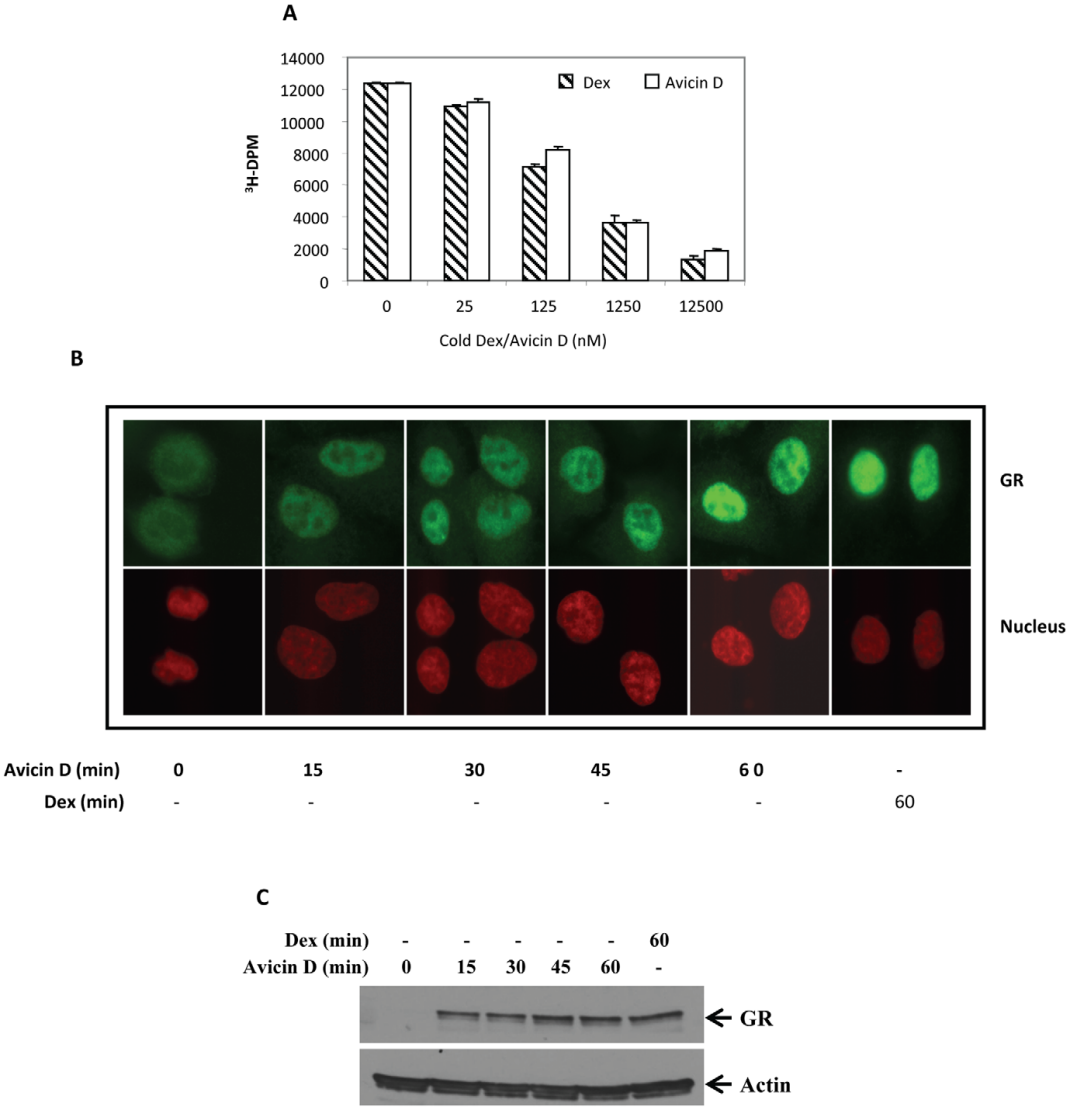
Avicin D binds to GR and translocates it into the nucleus. (A) A549 cells were incubated with 25 nM [^3^H] Dex in the presence or absence of 0–500 fold excess of cold Dex or cold avicin D. Following one hour incubation at 37°C, cells were lysed and radioactivity measured as described in the methods. (B & C) A549 cells were treated with avicin D (1 µM) for 0–60 min, or with Dex (1 µM) for 60 min at 37°C. (B) At the end of the incubation, cells were fixed and immunostained as described in the methods. (C) Western blot analysis of the nuclear extracts of treated cells was performed using anti- GR antibody as described in the methods. Actin expression was used as a loading control.

To determine if avicin D could interact with GR, we performed cold competition whole-cell binding assays in A549 cells, which are known to be high expressors of GR. Cells were incubated with 25 nM of ^3^H-Dex in the presence or absence of 0–500 fold of excess cold avicin D or cold Dex, for one hr. As shown in Fig. 2A, fivefold excess of cold avicin D inhibits the binding of labeled Dex by about 35%. When used at a 500 fold excess, avicin D inhibits the binding of labeled Dex by about 85%. Cold Dex was used as a positive control at concentrations similar to those of cold avicin D, and the inhibition induced by Dex and avicin D were comparable.

Inactive GR is held in the cytoplasm, bound to a protein complex which includes heat shock proteins. Ligand binding induces conformational changes in the GR, leading to its release from the protein complex, and translocation into the nucleus. Based on the observation that avicin D binds to GR, we next asked if this binding would lead to nuclear translocation of GR. A time course of avicin D treatment revealed a gradual entry of GR from the cytoplasm into the nucleus, using immunofluorescence (Fig. 2B). These findings were confirmed by western blot analysis of the nuclear extracts of avicin D treated cells (Fig. 2C). GR could be seen in the nuclear compartment within 15 min of treatment with avicin D, and at the end of 1 hr, the nuclear GR signal was comparable in both the avicin D and Dex treated A549 cells (Fig. 2B&C) Simultaneous changes in the cytoplasmic levels of GR were hard to visualize and quantify due to the abundance of inactivated GR in the cytoplasm (data not shown).

### Avicin D does not activate GR-driven transcription

**Figure 3:**
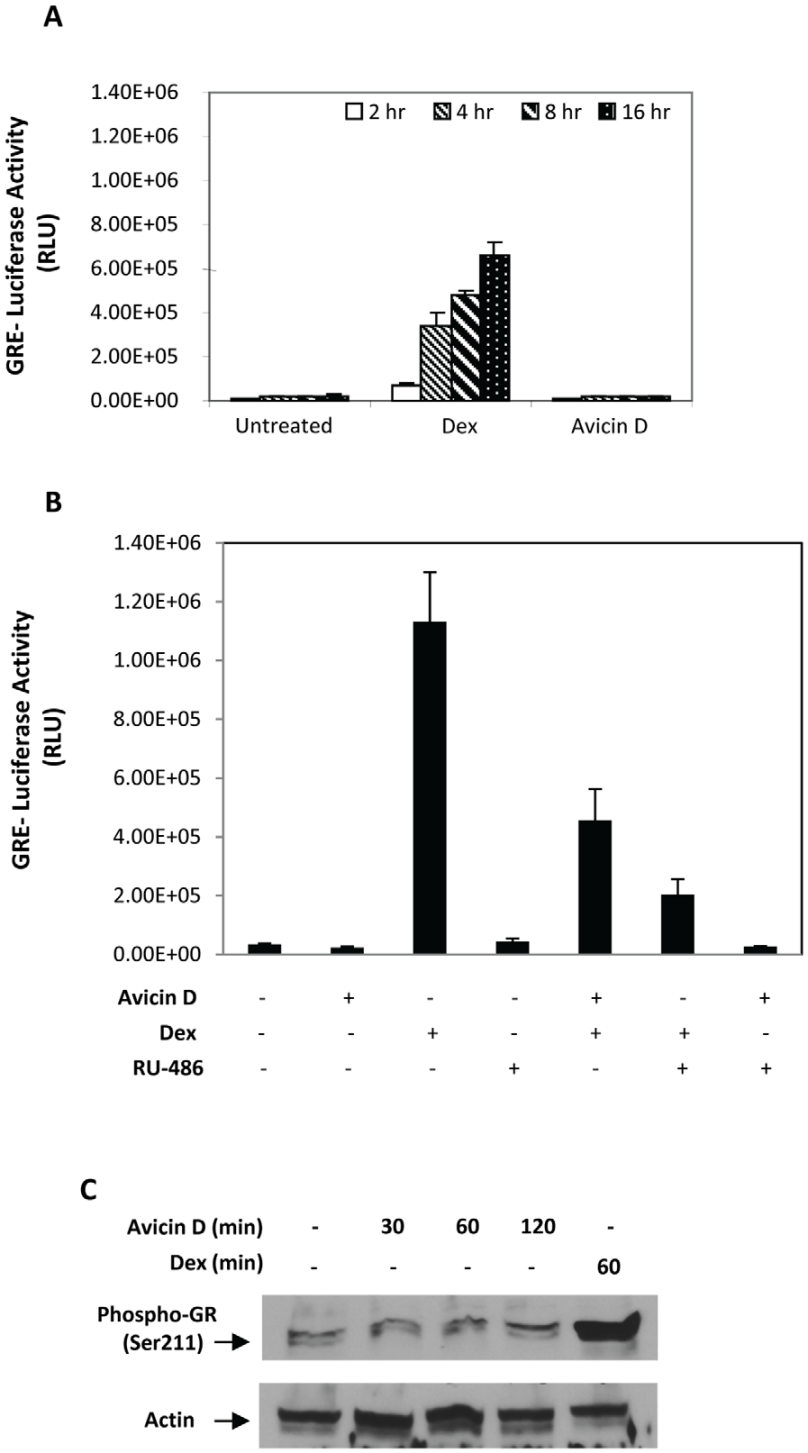
Effect of avicin D on GRE-dependent gene expression. (A) A549 cells transfected with p (GRE)_2_-50huIL6P-luc+ were treated with avicin D (1 µM) or Dex (1 µM) for 2–16 hrs. Luciferase activity was measured in cell lysates, and normalized for TK renilla luminescence as described in the methods. (B) Effect of avicin D and RU486 on Dex-induced luciferase activity. A549 cells transfected with p (GRE)_2_-50huIL6P-luc+ were pre-treated with avicin D (1 µM) or RU486 (1 µM) for 2 hrs, prior to being exposed to Dex (1 µM) or avicin D (1 µM) for 16 hrs. Luciferase activity was measured in cell lysates, and normalized for TK renilla luminescence as described in the methods. Luciferase activity has been expressed as relative luciferase units (RLU). (C) A549 cells were treated with Avicin D (1 µM) for 0–120 min or with Dex (1 µM) for 60 min. Western blot analysis of the nuclear extracts was performed using anti-phospho GR (Ser211) antibodies. Actin levels have been shown as a loading control.

Activated GR upon entering the nucleus binds to specific palindromic sequences, termed GREs, resulting in the transcriptional regulation of various genes. To study the effect of avicin D on the transactivation of GR, A549 cells transfected with p(GRE)_2_50hu.IL6P-luc+ construct were treated with avicin D or Dex. As shown in Fig. 3A, while Dex (1 µM) strongly induced luciferase activity in a time dependent manner, avicin D at the same dose had no effect even after a 16 hrs treatment. Higher concentrations (2 µM) of avicin D were also unable to induce luciferase activity (data not shown).

Based on the earlier finding that avicin D binds to GR, we next wanted to determine if avicin D can inhibit Dex induced transactivation of GR, by competing for binding to GR. RU486, a known GC antagonist was used as a control in this study. A549 cells transfected with p(GRE)_2_50hu.IL6P-luc+ construct were pre-treated with either Avicin D (1 µM) or RU486 (1 µM) for 2 hrs prior to being exposed to Dex (1 µM) for 16 hrs. By itself, avicin D once again was unable to induce any GR-dependent luciferase activity (Fig. 3B). However, pre-treatment with Avicin D, inhibited Dex induced luciferase activity by about 60%. RU486 at the same concentration decreased Dex induced luciferase activity by about 80%. These results demonstrate that avicin D though less potent than RU486, can block Dex-induced transactivation of GR. RU 486 pre-treatment had no significant effect on avicin D-induced luciferase activity, which was minimal to begin with (Fig. 3B).

Phosphorylation of specific serine residues, mostly in the DNA binding domain of GR have been shown to correlate with the receptor's transactivation activity. One such residue is Ser-211 in the N-terminus of GR, which becomes hyperphosphorylated upon stimulation of GR by an agonist [10]. A western blot analysis of the nuclear extracts from avicin D treated A549 cells showed that avicin D did not induce the phosphorylation of GR at Ser-211 (Fig. 3C). On the other hand, cells exposed to Dex under similar conditions displayed an increase in GR phosphorylation at Ser-211 (Fig. 3C). These findings taken together with the results shown in Figs. 2B and 2C, suggest that phosphorylation of GR at Ser-211 might not be a pre-requisite for its nuclear transport, instead might be crucial for mediating the transactivating effects of GR.

**Figure 4:**
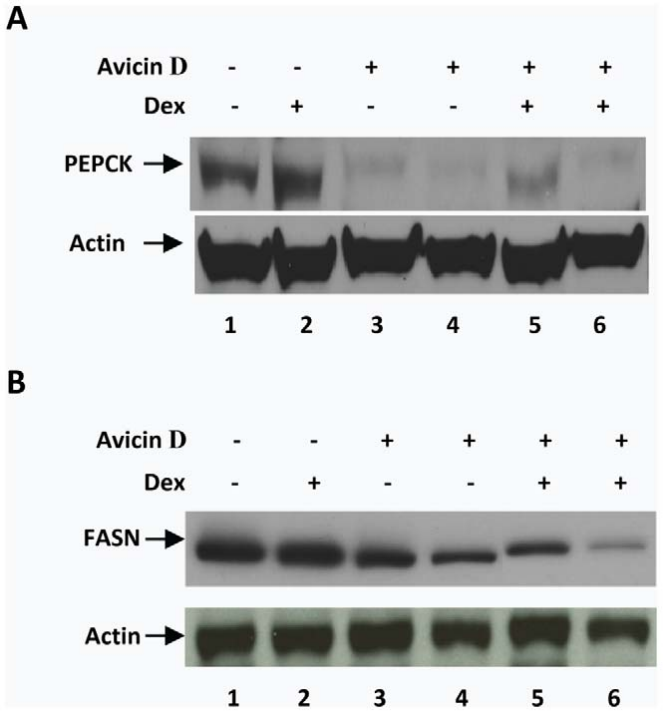
Effect of avicin D on PEPCK and FASN expression. Hep G2 cells were either untreated (lane 1), treated with Dex for 24 hrs (lane 2), or treated with avicin D for 48 (lane 3) and 72 (lane 4) hrs. Cells were also either co-treated with avicin D and Dex for 48 h (lane 5), or pretreated with avicin D (24 hrs), followed by a 24 hr treatment with Dex (lane 6). Avicin D and Dex were used at 1 µM each in all cases. Western blot analysis of total cell lysates was performed using anti-PEPCK (A) and anti-FASN (B) antibodies.

Based on the finding that avicin D does not turn on GR-driven transcription, we evaluated the expression of phosphoenolpyruvate carboxykinase (PEPCK) and fatty acid synthase (FASN), both GR-regulated proteins, in avicin D-treated cells. Since liver cells express high levels of PEPCK and FASN, we used HepG2, a hepatocarcinoma cell line which is a high expressor of GR (data not shown), for this analysis. While Dex treatment led to an increase in levels of PEPCK (Fig. 4A, lane 2), treatment with avicin D for 48 (Fig. 4A, lane 3) or 72 hrs (Fig. 4A, lane 4) resulted in a dramatic decrease in the basal levels of PEPCK. Avicin D also inhibited Dex-induced PEPCK, as seen in cells either co-treated with avicin D and Dex for 48 hrs (Fig. 4A, lane 5), or pre-treated with avicin D for 24 hrs followed by exposure to Dex for another 24 hrs (Fig. 4A, lane 6). Evaluation of FASN expression under similar experimental conditions yielded comparable results. Although Dex by itself was unable to induce additional FASN protein in these cells (Fig. 4B, lane 2), avicin D induces a significant decrease in levels of FASN when either given alone (Fig. 4B, lanes 3 and 4) or in combination with Dex (Fig. 4B, lanes 5 and 6). Consistent with the results shown in Fig. 3B, these findings suggest that avicin D can suppress GRE-mediated gene expression both directly and by competing with Dex for GR binding.

### Avicin D-induced apoptosis is not GR-dependent

**Figure 5:**
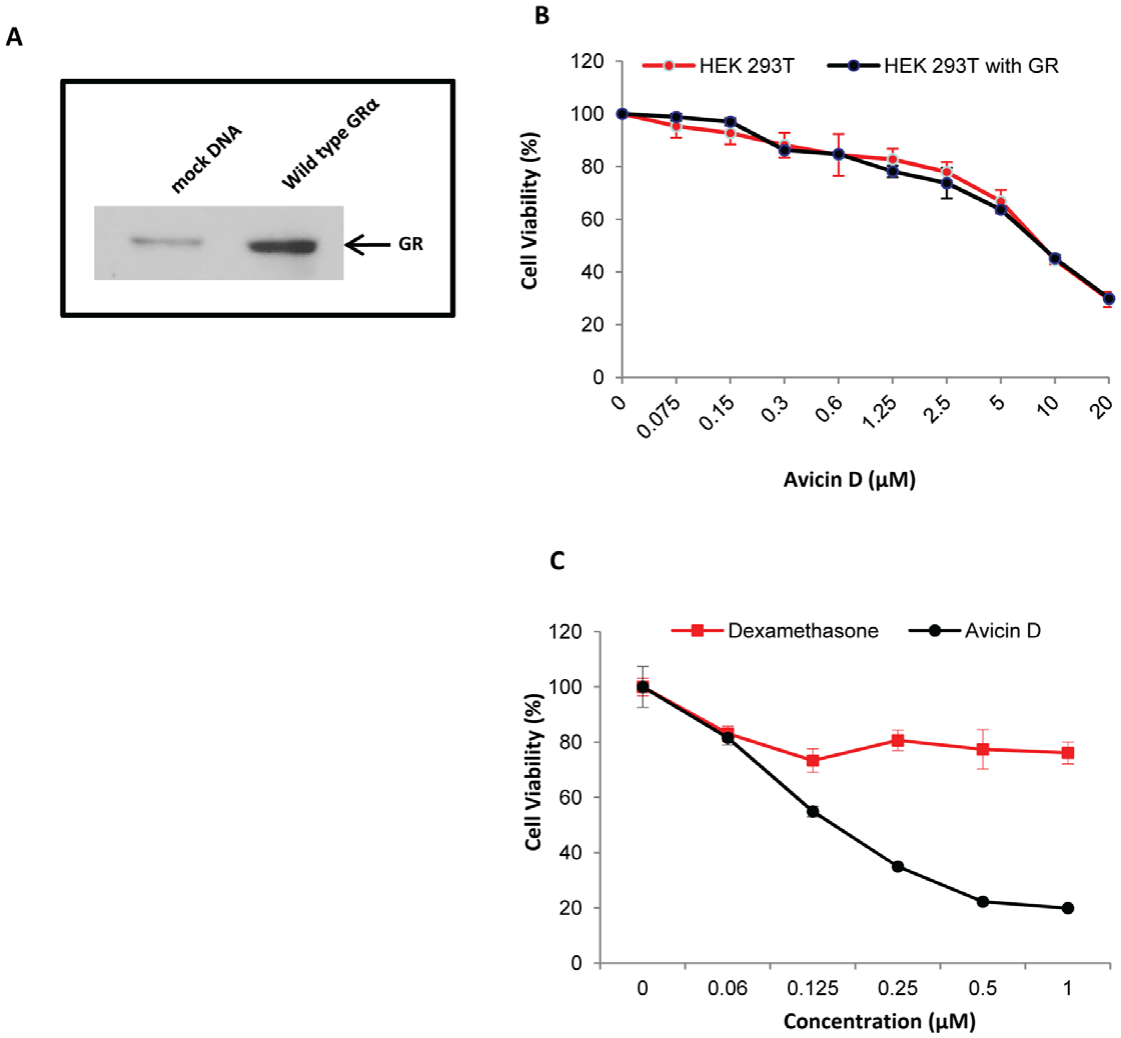
Avicin D induced cell killing is GR-independent. (A) Western blot comparing the GR expression in wild type HEK293T cells transfected with the mock DNA and plasmid containing the wild type GRα. (B) HEK293T cells transfected with mock DNA or wild type GRα were treated with different concentrations of avicin D for 72 hrs. Cell viability was measured using the MTT assay as described in the methods. (C) Jurkat cells were treated with different concentrations of avicin D or Dex for 72 hrs. At the end of 72 hrs, cell viability was evaluated using the MTT assay, as described in the methods.

One of the cellular effects regulated by GR-mediated gene activation is the induction of apoptosis [11]–[15]. In T-lymphocytes, the anti-inflammatory and immunomodulatory properties of GCs have been attributed to their ability to induce apoptosis [11], [12]. Based on the results presented so far, and given the fact that avicins are known to induce apoptosis in tumor cells [2], [16], we wanted to determine the role of GR in avicin-mediated apoptosis. To evaluate the involvement of GR signaling in avicin-induced cell death, we used HEK 293T cells which are very low expressors of GR (Fig. 5A). A GR over expressing version of these cells was generated by transfecting them with wild type GRα (Fig. 5A). In a cell viability assay, both the parental and GR over expressing HEK 293T cells were found to be equally responsive to avicin D (Fig. 5B), indicating that the avicin D-mediated cell killing is not dependent on the levels of GR expression. Jurkat, one of the most sensitive cell lines to avicin D-mediated cell death [16], lack endogenous GR [17], and are relatively resistant to Dex-induced cell killing (Fig. 5C). These results clearly show that (a) expression of GR is not a pre-requisite for cells to be sensitive to avicin-induced cell death and (b) signaling through the GR is not a key regulator of avicin-induced cell death.

### Avicin D inhibits TNF-induced NF-κB activation in a GR-dependent manner

**Figure 6:**
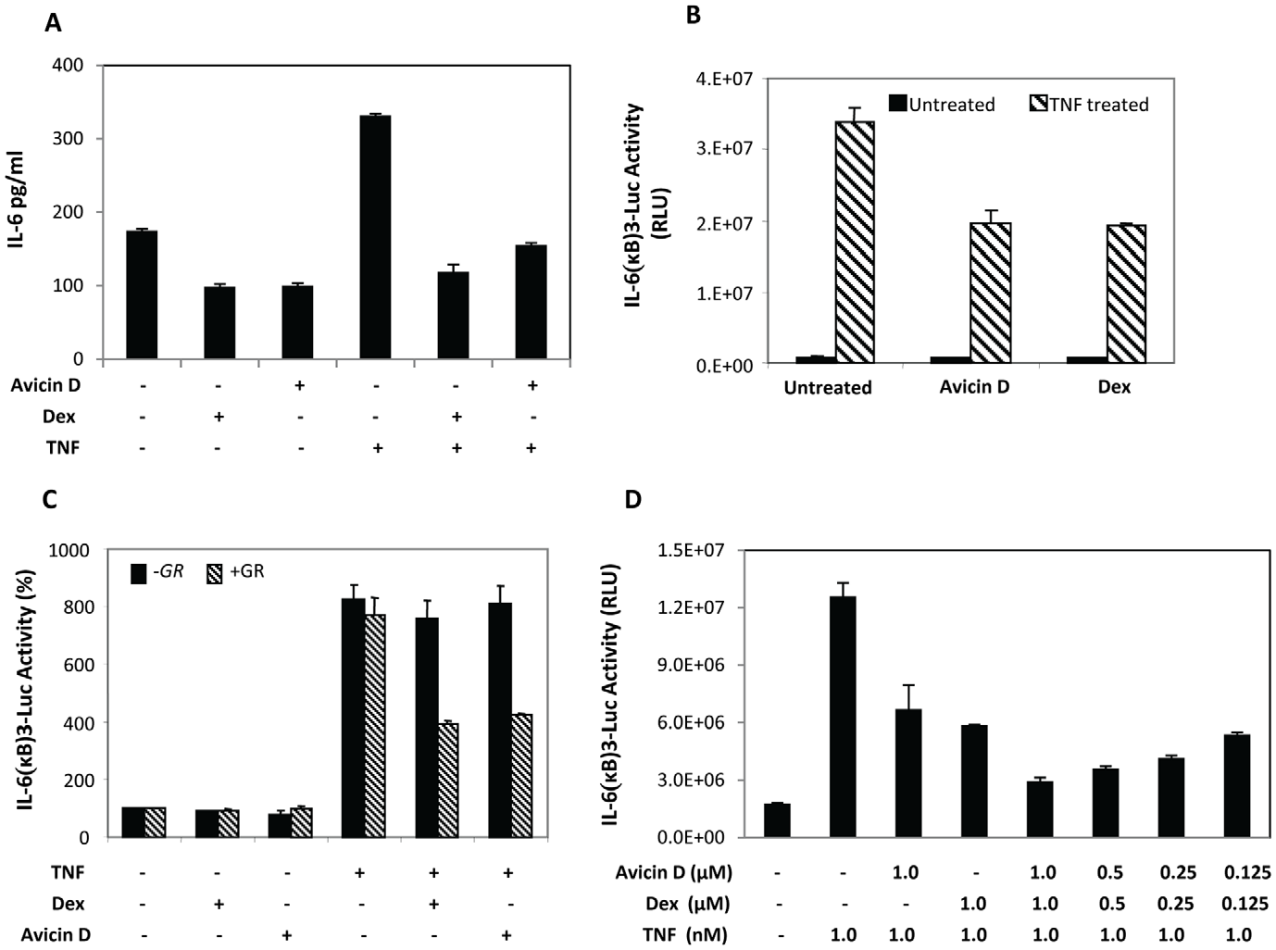
Avicin D inhibits activation of NF-κB. (A) A549 cells were pre-treated with 1 µM each of either avicin D or Dex for 24 hrs. Following the pre-treatment, cells were exposed to TNF (1 nM) for another 24 hrs. At the end of the TNF treatment, cell supernatants were collected. IL-6 levels were measured using an ELISA kit. (B) A549 cells transfected with p (IL6κB)3-50huIL6P-luc+ were either untreated or pre-treated with Avicin D/Dex (1 µM each) for 2 hrs, followed by a 4 hrs treatment with TNF (1 nM). Cell lysates were assayed for luciferase activity, and normalized for TK renilla luminescence as described in the methods. Luciferase activity has been expressed as relative luciferase units (RLU). (C) Normal and GR over expressing HEK 293T cells were transfected with 100 ng of p (IL6κB)3-50huIL6P-luc+ as described in the methods. Cells were next pre-treated treated with Avicin D/Dex (1 µM each) and treated with TNF (1 nM) as described for Fig. 6B. Luciferase activity was measured in cell lysates, and normalized for TK renilla luminescence as described in the methods. Luciferase activity has been expressed as % change over the untreated control, which in turn is taken as 100%. (D) A549 cells transfected with p(IL6κB)3-50huIL6P-luc+ were pre-treated with avicin D/Dex (1 µM each) either as single agents or in combinations for 2 hrs, followed by a 4 hrs treatment with TNF (1 nM). Cell lysates were assayed for luciferase activity and normalized for TK renilla luminescence as described in the methods. Luciferase activity has been expressed as relative luciferase units (RLU).

The anti-inflammatory effects of GCs are mediated via the transrepressive action of GR, which involves its interaction and inhibition of other proteins, mostly transcription factors. One such transcription factor is the NF-κB. To demonstrate the effect of avicin D on NF-κB signaling, we first evaluated the levels of IL-6, an NF-κB regulated, pro-inflammatory endogenous cytokine in avicin treated cells. A549 cells were treated with TNF (1 nM) for 24 hrs to induce IL-6 production. Prior to being exposed to TNF, cells were either left untreated or pre-treated with avicin D (1 µM)/Dex (1 µM) for 24 hrs. Fig. 6A shows the levels of IL-6 measured in the supernatants of these cultures at the end of 48 hrs. Treatment with both avicin D and Dex resulted in a significant decrease in the levels of both basal as well as TNF-induced IL-6. To further confirm the effects of avicin D on activation of NF-κB, we studied TNF-induced NF-κB activity in A549 cells, transfected with p(IL6κB)_3_50hu.IL6Pluc+, an NF-κB-dependent human IL-6 promoter construct. As shown in Fig. 6B, TNF induces a dramatic increase in NF-κB activity, which is measured by the increase in IL-6(κB)3- luciferase activity. Treatment of cells with avicin D or Dex alone does not have any significant effect on the basal levels of NF-κB activity. However, pretreatment of these cells with avicin D (1 µM)/Dex (1 µM) abrogates the TNF-induced NF-κB activity significantly. These results are consistent with our earlier findings that show the avicins inhibit activation of NF-κB [5].

Next, to determine the involvement of GR in avicin-induced inhibition of NF-κB, we used HEK 293 T cells, which express negligible levels of endogenous GR (Fig. 5A). We evaluated the effect of avicin D and Dex on activation of NF-κB-driven luciferase activity in wild type and GR over expressing HEK 293 T cells. As shown in Fig. 6C, avicin D or Dex had no effect on the TNF-induced luciferase activity in wild type HEK 293T (-GR) cells. However, TNF-induced activation of p(IL6-κB)_3_50hu.IL6P-Luc was inhibited by avicin D/Dex in HEK 293T cells transfected with GR (+GR) (Fig. 6C), indicating that the presence of GR is required for avicin D and Dex to downregulate the activation of NF-κB.

Since both avicin D and Dex bind to GR, we next wanted to determine the effect of avicin D in combination with Dex, on inhibition of NF-κB. A549 cells transfected with p(IL6κB)_3_50hu.IL6Pluc+, were pretreated with combinations of avicin D and Dex, before being exposed to TNF. As shown in Fig. 6D, avicin D (1 µM) or Dex (1 µM) alone reduced TNF-induced NF-κB activity by about 50%. A combination of 1 µM each of avicin D and Dex was more effective than either agent by themselves, suggesting an additive effect. Combinations of lower concentrations upto 0.125 µM, were also effective in inhibiting TNF-induced NF-κB activation (Fig. 6D). However, a gradual reversal in effectiveness could be seen as the concentrations of Avicin D/Dex kept decreasing, suggesting that the two agents might not have a synergistic effect.

### The ligand and DNA-binding domains of GR are critical of avicin-mediated inhibition of NF-κB

**Figure 7:**
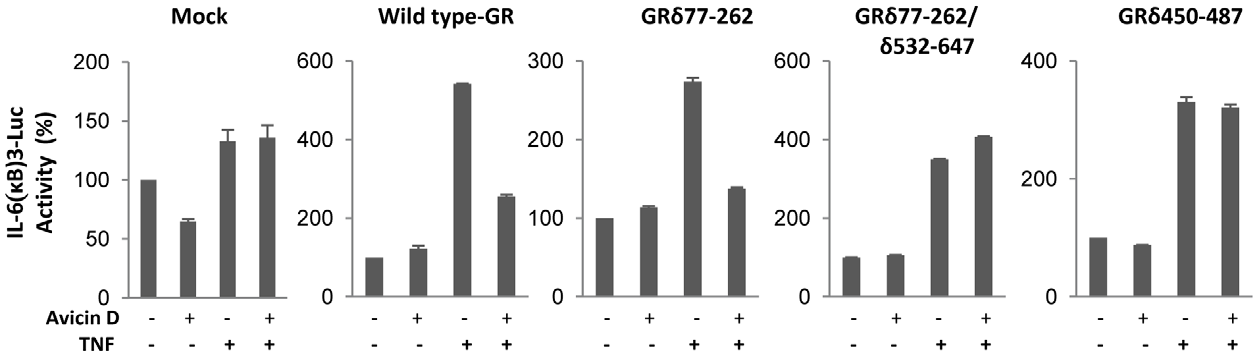
Role of different GR domains in avicin D-mediated inhibition of NF-κB activity. HEK 293T cells were transiently transfected with mock DNA, or plasmids carrying either wild type GRα or deletion variants of GR. After transfection, cells were pre-treated with Avicin D (1 µM) for 2 hrs, followed by a 4 hrs treatment with TNF (1 nM). Luciferase activity was measured in the cell lysates, and normalized for TK renilla luminescence as described in the methods. Luciferase activity has been expressed as % change over the untreated control, which in turn is taken as 100%.

The human GR has been shown to be composed of a series of discrete functional domains [18], [19]. In order to determine which domain of the GR molecule is involved in avicin-induced inhibition of NF-κB, we used different deletion mutants of the GR. HEK 293T cells were transfected with mock DNA, or with plasmids carrying either the wild type GR or GR with deletions in its different domains. Effect of avicin D on TNF-induced activation of p(IL6-κB)_3_50hu.IL6P-Luc was then studied in these transfected cells. The results shown in Fig. 7, demonstrate that mutations in the DNA- and ligand-binding domains (GRδ77-262/δ532-647 and GRδ450-487 respectively) reverse the avicin-induced inhibition of NF-κB activity, while mutations in the N-terminal part of the GR (GRδ77-262) appear to have no such effect. These findings suggest that while the N-terminal appears to be dispensable, both the intact DNA- and ligand binding domains are critical for the avicin D to exert its transrepressive effects.

### Modeling of avicin D-GR interaction

**Figure 8:**
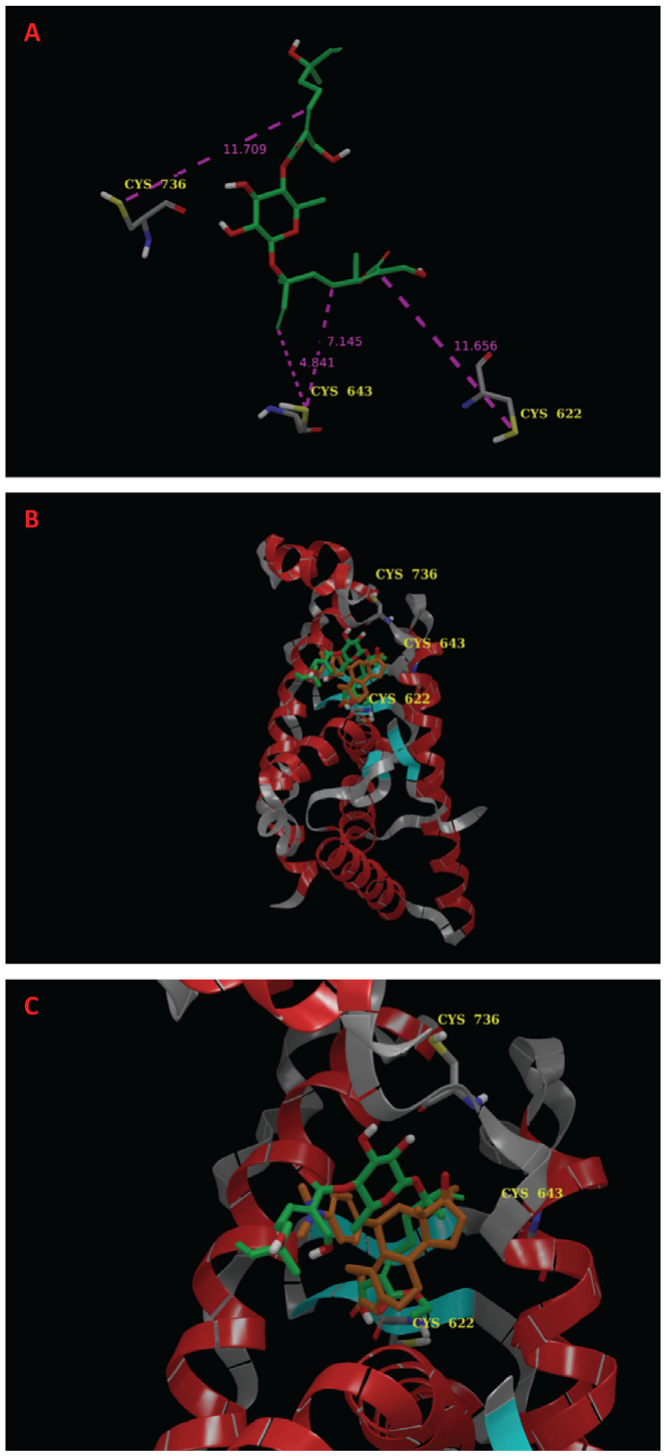
A model of avicin D-GR interaction. The Avicin-D warhead was docked into the crystal structure of RU-486 bound to the antagonist form of glucocorticoid (pdb code: 1NHZ) as described in the methods. (A) Distances between the Sg of three cystein residues and the olefin groups of Avicin-D warhead (green carbon atoms). (B) Ribbon structure of 1NHZ with RU-486 (brown carbon atoms) and the model of the Avicin-D warhead (green carbon atoms). (C) Same as (B) except focused on the binding pocket of RU-486.

Based on all the findings described above, we next attempted to model the interaction between avicin D and the GR (Fig. 8). The warhead portion of Avicin-D (molecular fragment 2 shown in Figure 1C) was docked into two separate crystal structures for GR: 1M2Z and 1NHZ. The 1M2Z structure is the agonist version of the ligand binding domain (co-crystallized with dexamethasone) while 1NHZ is the antagonist form of the receptor (co-crystallized with RU-486). No energetically favorable poses were found for the 1M2Z structure because Avicin-D is too large to dock into the compact agonist binding site of GR. In the antagonist 1NHZ crystal structure, the warhead docks closest to Cys-643 and is relatively close to the more solvent exposed Cys-736 and Cys-622 amino acids. Figure 8 shows the spatial orientation of all cysteine residues from the 1NHZ crystal structure relative to the docked warhead. Two olefin groups of the warhead are close to Cys-643 and therefore could undergo thioesterification, an avicin-induced modification of cysteine residues which has been previously reported in the bacterial system, with Oxy R protein [20]. Additionally, the cystein sulfhydryl groups could form covalent complexes with the olefins. Further studies are required to evaluate such a modification.

## Discussion

In this study we report “glucocorticoid-like” properties of avicin D, a triterpene isolated from the seed pods of an Australian tree called *Acacia Victoria*. Like the GCs, the avicins have evolved from the ancient “hopanoids”, which could explain the resemblance between the core structures of these two classes of agents. Avicins which were first identified as inducers of apoptosis in tumor cells, have been shown to be anti-inflammatory and regulate cellular metabolism [3]–[5], [7], both effects attributed to GCs as well. These similar biological properties combined with the structural resemblance prompted us to compare the signaling mechanisms of avicins with those of the GCs. GCs are known to act by activating the GR, which is otherwise held in an inert state bound to intracellular chaperones [21]. Subsequent to ligand binding, activation of GR involves, (a) its nuclear translocation, (b) transactivation or binding to GC-responsive element (GRE) to regulate gene expression, and (c) transrepression or interaction with other transcription factors to facilitate or hinder their action [22], [23]. In this study we show that avicin D competes with Dex to bind to GR, and translocates it into the nucleus. Avicin D-induced nuclear translocation of GR does not lead to induction of GR-driven genes as measured by luciferase activity. Consistent with this observation was the finding that PEPCK and FASN, two GRE-regulated proteins were down regulated in response to avicin D treatment. Also, induction of cell death, which is believed to be regulated by transactivation of GR [13]–[15], was found to be non-dependent on GR activation in avicin treated cells. In an earlier study avicins have been shown to be (a) more potent inducers of cell death compared to Dex, using a panel of GC-responsive myeloma cells, and (b) capable of sensitizing primary multiple myeloma cells to Dex, even in the presence of bone marrow stromal cells (BMSCs), which are known to attenuate the cytotoxic effects of Dex [8]. These results can be explained by the possibility that avicin-induced cell death is independent of GR. Activation of GRE-regulated genes also involves positive combinatorial responses between GR and a variety of transcription factors including members of the signal transducer and activator of transcription (STAT) family such as STAT1, STAT3, and STAT5 [24]. We have previously demonstrated the dephosphorylation of Stat3 and down regulation of its transcriptional activity in response to avicin treatment in myeloma cells [25]. Whether the lack of GR-induced transactivation following avicin treatment could be related to avicins' effects on Stat3, needs to be evaluated.

Although unable to induce transcription of GRE-regulated genes, avicin-induced nuclear translocation of GR does result in suppression of TNF-induced activation of NF-κB in A549 cells, suggesting that the transrepressive function of GR is intact following avicin treatment. Using HEK 293 cells, which lack GR expression, we show that the suppression of NF-κB is GR-dependent, with the ligand and DNA binding domains of GR being crucial. However, our earlier studies showing decreased NF-κB activity in avicin-treated Jurkat cells, do point to an additional mechanism for avicin-mediated inhibition of NF-κB, in cells that are poor expressors of GR. In Jurkat cells, avicin-mediated inhibition of NF-κB was found to be redox dependent, by modifying critical cysteines on in the p65 subunit of NF-κB and thereby blocking its binding to DNA [5]. Though activation of NF-κB is known to play a role in the regulation of cell survival and apoptosis [26], [27], avicin-induced apoptosis does not appear to be under the control of NF-κB. Wild type and GR overexpressing HEK 293 cells have been found to be equally responsive to avicin D-induced cell kill, though avicin-induced inhibition of NF-κB is seen only upon expression of GR in these cells. This is not surprising based on our earlier studies which have demonstrated that avicins primarily target the mitochondrial bioenergetics to initiate apoptosis [2]–[4]. Kinetically, the effects on mitochondria, and activation of the apoptotic cascade appear before we see an avicin-mediated inhibition of NF-κB activity [2], [5], suggesting that inhibition of NF-κB is not the key regulator of avicin-induced apoptosis. Like NF-κB, other signaling pathways such as the PI3K/Akt, and Stat3 and their downstream targets like c-myc, survivin, Bcl2, and Mcl 1, all known to be regulators of cell survival and apoptosis have also been shown to be downregulated in avicin treated cells, though at a later time point [16], [25]. We therefore believe that avicin-mediated cell death gets triggered by perturbation of the mitochondria, and can subsequently be under the regulation of these signaling pathways.

Most of the undesirable side effects of GCs such as central adiposity, dyslipidaemia, skeletal muscle wasting, osteoporosis, insulin resistance, glucose tolerance and diabetes have been attributed to the transactivation arm of GR [28]–[30]. On the other hand, interfering with transcription factors such as NF-κB, and AP-1, with or without DNA binding, and repressing the transcription of downstream inflammatory genes has been considered to be the key mechanisms underlying the anti-inflammatory properties of GCs [28]–[30]. However recent studies have suggested that transactivation underlies some of the anti-inflammatory effects of GCs [31], [32]. Using microarray analysis of fluticasone treated human monocytes, Ehrchen et al., have demonstrated that more than 100 genes were induced compared to about 40 genes that were down-regulated, suggesting that transactivation prevails over transrepression [31]. Consistent with these findings is the recent questioning of the concept of “a dissociated ligand” [33]–[34], which was based on the assumption that GR mediated transactivation and transrepression had clearly distinguishable effects [35]–[37]. GR-induced genes (transactivation) such as DUSP1, GILZ, IκBα, SOCS1, IL-4, IL-10, tristetraprolin, decoy IL1 receptor type II, and TGFβ have been reported to contribute to GC's anti-inflammatory effects [34], [38]. MKP-1, a dual specificity phosphatase 1, that dephosphorylates and inactivates p38 MAPK, which in turn induces the expression of inflammatory transcription factors such as ATF-1, ATF-2 and AP-1 [39], [40], is strongly induced by GCs. Induction of MKP-1 leads to the transcriptional repression of inflammatory genes such as E-selectin [41]. Likewise, GR-mediated transrepression has also been known to underlie some of the GC-mediated side effects such as the HPA-axis suppression [42], [43]. These studies clearly indicate an overlap between the mechanisms that regulate the beneficial and deleterious effects of GCs.

Modeling of avicin D-GR interaction has shown that avicin-D is too large to dock to the agonist form of the glucocorticoid ligand binding domain. However, in the antagonist 1NHZ crystal structure, the warhead docks close to Cys-643. The avicin molecule contains Michael acceptor sites and reactive oxyesters [6], which have been shown to be involved in the transesterification of OxyR, a bacterial transcription factor OxyR, at a critical cysteine residue, leading to the activation of OxyR [20]. The effect of avicin D binding to Cys-643, on the GR activity remains to be studied. It is also possible, that the binding hooks avicin D to the GR, after which the side chain probably is clipped off, releasing the core GC-like structure of avicin D. Further studies are required to evaluate the exact nature of this interaction between GR and avicin D.

In conclusion, avicins which appear to have evolved as a stress regulatory molecule in an Acacia desert tree, clearly has profound biological effects on human cells, much like many plant-derived compounds that play important therapeutic role in clinical medicine [44]–[46]. The results reported in this paper also support a link between avicins' ability to regulate cellular stress [6], [20], as well as organismal stress via the glucocorticoid axis. To get an idea of avicin's effects in a more physiological system we chose to study its effect on the process of adipocyte differentiation. Differentiation of pre-adipocytes into adipocytes was induced using Dex and we evaluated the effect of avicin D on this process. Adipose redistribution, one of the undesirable results of GC treatment is a result of GR-induced transactivation of genes involved in adipocyte differentiation [47]. These studies and their results have been described in a manuscript, submitted for publication.

Avicin D has been approved by the United States Food and Drug Administration for phase I studies in human cancer patients. Based on the performance of some of the other dissociated GR ligands [37] *in vivo*, the effects of avicins *in vivo* are hard to predict and may depend on the dose and schedule of administration.

## References

[1] Jayatilake GS, Freeberg DR, Liu Z, Richheimer SL, Blake Nieto ME (2003). Isolation and structures of avicins D and G: in vitro tumor-inhibitory saponins derived from Acacia victoriae.. J Nat Prod.

[2] Haridas V, Higuchi M, Jayatilake GS, Bailey D, Mujoo K (2001). Avicins: triterpenoid saponins from Acacia victoriae (Bentham) induce apoptosis by mitochondrial perturbation.. Proc Natl Acad Sci USA.

[3] Haridas V, Li X, Mizumachi T, Higuchi M, Lemeshko VV (2007). Avicins, a novel plant-derived metabolite lowers energy metabolism in tumor cells by targeting the outer mitochondrial membrane.. Mitochondrion.

[4] Xu ZX, Liang J, Haridas V, Gaikwad A, Connolly FP (2007). A plant triterpenoid, avicin D, induces autophagy by activation of AMP-activated protein kinase.. Cell Death Differ.

[5] Haridas V, Arntzen CJ, Gutterman JU (2001). Avicins, a family of triterpenoid saponins from Acacia victoriae (Bentham), inhibit activation of nuclear factor-kappa B by inhibiting both its nuclear localization and ability to bind DNA.. Proc Natl Acad Sci USA.

[6] Haridas V, Hanausek M, Nishimura G, Soehnge H, Gaikwad A (2004). Triterpenoid electrophiles (avicins) activate the innate stress response by redox regulation of a gene battery.. J Clin Invest.

[7] Hanausek M, Ganesh P, Walaszek Z, Arntzen CJ, Slaga TJ (2001). Avicins, a family of triterpenoid saponins from Acacia victoriae (Bentham), suppress H-ras mutations and aneuploidy in a murine skin carcinogenesis model.. Proc Natl Acad Sci USA.

[8] Mitsiades N, McMullan CJ, Poulaki V, Negri J, Gutterman JU (2004). Avicins: a novel class of anti-myeloma agents.. Blood.

[9] Bamberger CM, Bamberger AM, de Castro M, Chrousos GP (1995). Glucocorticoid receptor beta, a potential endogenous inhibitor of glucocorticoid action in humans.. J Clin Invest.

[10] Wang Z, Frederick J, Garabedian MJ (2002). Deciphering the phosphorylation “code” of the glucocorticoid receptor in vivo.. J Biol Chem.

[11] Brewer JA, Kanagawa O, Sleckman BP, Muglia LJ (2002). Thymocyte apoptosis induced by T cell activation is mediated by glucocorticoids in vivo.. J Immunol.

[12] Herold MJ, McPherson KG, Reichardt HM (2006). Glucocorticoids in T cell apoptosis and function.. Cell Mol Life Sci.

[13] Tuckermann JP, Kleiman A, McPherson KG, Reichardt HM (2005). Molecular mechanisms of glucocorticoids in the control of inflammation and lymphocyte apoptosis.. Crit Rev Clin Lab Sci.

[14] Distelhorst CW (2002). Recent insights into the mechanism of glucocorticosteroid-induced apoptosis.. Cell Death Differ.

[15] Frankfurt O, Rosen ST (2004). Mechanisms of glucocorticoid-induced apoptosis in hematologic malignancies: updates.. Curr Opin Oncol.

[16] Mujoo K, Haridas V, Hoffmann JJ, Wächter GA, Hutter LK (2001). Triterpenoid saponins from Acacia victoriae (Bentham) decrease tumor cell proliferation and induce apoptosis.. Cancer Res.

[17] Helmberg A, Auphan N, Caelles C, Karin M (1995). Glucocorticoid-induced apoptosis of human leukemic cells is caused by the repressive function of the glucocorticoid receptor.. EMBO J.

[18] Giguère V, Hollenberg SM, Rosenfeld MG, Evans RM (1986). Functional domains of the human glucocorticoid receptor.. Cell.

[19] Hollenberg SM, Giguere V, Segui P, Evans RM (1987). Co localization of DNA-binding and transcriptional activation functions in the human glucocorticoid receptor.. Cell.

[20] Haridas V, Kim SO, Nishimura G, Hausladen A, Stamler JS (2005). Avicinylation (thioesterification): a protein modification that can regulate the response to oxidative and nitrosative stress.. Proc Natl Acad Sci USA.

[21] Pratt WB, Gehring U, Toft DO (1996). Molecular chaperoning of steroid hormone receptors.. EXS.

[22] Barnes PJ (2006). How corticosteroids control inflammation: Quintiles Prize Lecture 2005.. Br J Pharmacol.

[23] Rhen T, Cidlowsky JA (2005). Anti-inflammatory action of glucocorticoids: new mechanisms for old drugs.. N Engl J Med.

[24] Kordula T, Travis J (1996). The role of Stat and C/EBP transcription factors in the synergistic activation of rat serine protease inhibitor-3 gene by interleukin-6 and dexamethasone.. Biochem J.

[25] Haridas V, Nishimura G, Xu ZX, Connolly F, Hanausek M (2009). Avicin D: a protein reactive plant isoprenoid dephosphorylates Stat 3 by regulating both kinase and phosphatase activities.. PLoS One.

[26] Karin M (2006). Nuclear factor-kappa B in cancer development and progression.. Nature.

[27] Dutta J, Fan Y, Gupta N, Fan G, Gelinas C (2006). Current insights into the regulation of programmed cell death by NF-kappa B.. Oncogene.

[28] De Bosscher K, Vanden Berghe W, Haegeman G (2003). The interplay between the glucocorticoid receptor and nuclear factor-kappa B or activator protein-1: molecular mechanisms for gene repression.. Endocr Rev.

[29] Reichardt HM, Tuckermann JP, Bauer A, Schütz G (2000). Molecular genetic dissection of glucocorticoid receptor function in vivo.. Z Rheumatol.

[30] Schäcke H, Döcke WD, Asadullah K (2002). Mechanisms involved in the side effects of glucocorticoids.. Pharmacol Ther.

[31] Ehrchen J, Steinmüller L, Barczyk K, Tenbrock K, Nacken W (2007). Glucocorticoids induce differentiation of a specifically activated, anti-inflammatory subtype of human monocytes.. Blood.

[32] Surjit M, Ganti KP, Mukherji A, Ye T, Hua G (2011). Widespread negative response elements mediate direct repression by agonist- liganded glucocorticoid receptor.. Cell.

[33] Belvisi MG, Wicks SL, Battram CH, Bottoms SE, Redford JE (2001). Therapeutic benefit of a dissociated glucocorticoid and the relevance of in vitro separation of transrepression from transactivation activity.. J Immunol.

[34] Newton R, Holden NS (2007). Separating transrepression and transactivation: a distressing divorce for the glucocorticoid receptor?. Mol Pharmacol.

[35] Vanden Berghe W, Francesconi E, De Bosscher K, Resche-Rigon M, Haegeman G (1999). Dissociated glucocorticoids with anti-inflammatory potential represses interleukin-6 gene expression by a nuclear factor-kappa B-dependent mechanism.. Mol Pharmacol.

[36] De Bosscher K, Beck IM, Haegeman G (2010). Classic glucocorticoids versus non-steroidal glucocorticoid receptor modulators: survival of the fittest regulator of the immune system?. Brain Behav Immun.

[37] De Bosscher K (2010). Selective Glucocorticoid Receptor modulators.. J Steroid Biochem Mol Biol.

[38] Clark AR (2007). Anti-inflammatory functions of glucocorticoid-induced genes.. Mol Cell Endocrinol.

[39] Wesselborg S, Bauer MK, Vogt M, Schmitz ML, Schulze-Osthoff K (1997). Activation of transcription factor NF-kappa B and p38 mitogen-activated protein kinase is mediated by distinct and separate stress effector pathways.. J Biol Chem.

[40] Newton R, Holden N (2003). Inhibitors of p38 mitogen-activated protein kinase: potential as anti-inflammatory agents in asthma?. BioDrugs.

[41] Fürst R, Schroeder T, Eilken HM, Bubik MF, Kiemer AK (2007). MAPK phosphatase-1 represents a novel anti-inflammatory target of glucocorticoids in the human endothelium.. FASEB J.

[42] Otten U, Baumann JB, Girard J (1979). Stimulation of the pituitary-adrenocortical axis by nerve growth factor.. Nature.

[43] Zora JA, Zimmerman D, Carey TL, O'Connell EJ, Yunginger JW (1986). Hypothalamic-pituitary-adrenal axis suppression after short-term, high-dose glucocorticoid therapy in children with asthma.. J Allergy Clin Immunol.

[44] Paterson I, Anderson EA (2005). The renaissance of natural products as drug candidates.. Science.

[45] Johnson IS (1992). Drugs from Third World plants.. Science.

[46] Polizzi D, Pratesi G, Tortoreto M, Supino R, Riva A (1999). A novel taxane with improved tolerability and therapeutic activity in a panel of human tumor xenografts.. Cancer Research.

[47] Masuzaki H, Paterson J, Shinyama H, Morton NM, Mullins JJ (2001). A transgenic model of visceral obesity and the metabolic syndrome.. Science.

